# Association of plasma endocan levels with metabolic parameters and predictive value of endocan for the development of complications in patients with type 2 diabetes mellitus: An observational study

**DOI:** 10.17305/bb.2024.11512

**Published:** 2025-12-18

**Authors:** Kenana Ljuca, Mensura Aščerić, Olivera Batić-Mujanović, Svjetlana Loga-Zec, Nadina Ljuca, Emir Bećirović, Samir Bejić, Predrag Jovanović, Minela Bećirović

**Affiliations:** 1 Department of Gynecology and Obstetrics, University Clinical Center Ljubljana, Ljubljana, Slovenia; 2 Health Center of Sarajevo Canton, Sarajevo, Bosnia and Herzegovina; 3 School of Medicine, University of Tuzla, Tuzla, Bosnia and Herzegovina; 4 Department of Pharmacology, School of Medicine, University of Tuzla, Tuzla, Bosnia and Herzegovina; 5 Department of Family Medicine, Health Center of Tuzla, Tuzla, Bosnia and Herzegovina; 6 Department of Pharmacology, School of Medicine, University of Sarajevo, Sarajevo, Bosnia and Herzegovina; 7 Internal Medicine Clinic, University Clinical Center Tuzla, Tuzla, Bosnia and Herzegovina

**Keywords:** Endocan, diabetes mellitus type 2, DMT2, metabolic parameters, predictive value

## Abstract

The aim of the current research was to investigate the association between plasma endocan levels and metabolic control parameters, as well as to evaluate its predictive value for clinical complications in patients with type 2 diabetes mellitus (DMT2). A total of 100 DMT2 patients participated in this prospective observational study. Plasma endocan levels were significantly elevated in DMT2 patients with HbA1c > 7% (1.38 ± 0.33 vs 0.68 ± 0.23 ng/mL; *P* < 0.0001), compared to patients with HbA1c ≤ 7%. Patients with plasma endocan concentrations >1.10 ng/mL (median value of 1.10 ng/mL) demonstrated significantly higher levels of metabolic parameters: body mass index (BMI), HbA1c (%), fasting glucose level, LDL cholesterol, total cholesterol, triglycerides, along with significantly lower HDL cholesterol levels. Furthermore, patients with plasma endocan levels **>**1.10 ng/mL were found to have an increased risk for the following complications: retinopathy (relative risk [RR]: 2.7500; 95% confidence interval [CI]: 1.2150–6.2244; *P* ═ 0.0152, nephropathy (RR: 2.0952; 95% CI: 1.2294–3.5710; *P* ═ 0.0065), neuropathy (RR: 1.9945; 95% CI: 1.2025–3.3081; *P* ═ 0.0075), angina pectoris (RR: 2.4881; 95% CI: 1.0865–5.6979; *P* **=** 0.0311, hypertension (RR: 1.1372; 95% CI: 1.0060–1.2856; *P* **=** 0.0398), cardiomyopathy (RR: 2.6190; 95% CI: 1.1507–5.9612; *P* **=** 0.0218), myocardial infarction (RR: 9.4286; 95% CI: 1.2742–69.7697; *P* **=** 0.0280) and stroke (RR: 4.4638; 95% CI: 1.3765–14.4758; *P* **=** 0.0127). Correlation analysis revealed that plasma endocan levels were positively correlated with HbA1c (%) (*r* ═ 0.856, *P* < 0.0001), fasting glucose level (*r* ═ 0.631, *P* < 0.0001), LDL (*r* ═ 0.347, *P* ═ 0.0004), cholesterol (*r* ═ 0.282, *P* ═ 0.0045), and triglycerides (*r* ═ 0.366, *P* ═ 0.0002). Conversely, plasma endocan levels were negatively correlated with HDL cholesterol (*r* ═ −0.429, *P* < 0.0001). In conclusion, higher plasma endocan levels were strongly associated with poor metabolic control in DMT2 patients and exhibited significant predictive value for both microvascular and macrovascular complications.

## Introduction

Endocan is a proteoglycan expressed and produced by the vascular endothelium. It stimulates the secretion of pro-inflammatory cytokines, promotes white blood cell migration, and increases vascular permeability. Due to its influence on inflammatory and vasculoprotective mechanisms, endocan is thought to play a key role in endothelial dysfunction [1, 2]. In diseases marked by endothelial damage and neovascularization, serum or plasma endocan levels tend to increase [3, 4]. Diabetes mellitus (DM) is a major global health issue. Despite advancements in diagnostic and therapeutic strategies, the prevalence of DM remains high, with type 2 DM (DMT2) accounting for 90% of all cases [5]. According to the International Diabetes Federation, the global prevalence of diabetes in adults was 10.5% in 2021 and is projected to rise to 12.2% by 2045 [6]. Both microvascular and macrovascular complications are common across all types of DM, driven primarily by endothelial dysfunction [7]. Endocan has emerged as a novel biomarker for endothelial dysfunction in DM. Several studies have reported elevated serum or plasma endocan levels in patients with type 1 (DMT1) [8] and DMT2 [9–13]. However, two studies observed lower endocan levels in DMT2 patients compared to healthy individuals [14, 15]. These conflicting findings suggest that the pathophysiological role and clinical relevance of endocan in DM—particularly in relation to different disease stages, metabolic control, and its predictive value for complications—remain unclear. This study aims to evaluate the association between plasma endocan levels and metabolic parameters and assess their predictive value for microvascular and macrovascular complications in DMT2 patients.

## Materials and methods

### Patients and methods

#### Patients

This prospective consecutive cohort observational study included 100 patients with DMT2 who were treated at the Health Center of Sarajevo Canton, Bosnia and Herzegovina, during November 2023. The inclusion criteria for this research were: a) patients with DMT2 for ≥5 years and b) age ≥50 years. The exclusion criteria included: a) newly diagnosed DMT2 patients, b) patients with type 1 DM (DMT1), c) DMT2 patients younger than 50 years, d) patients with DMT2 < 5 years, e) individuals with prediabetes, and f) patients with malignant disease.

**Figure 1.**
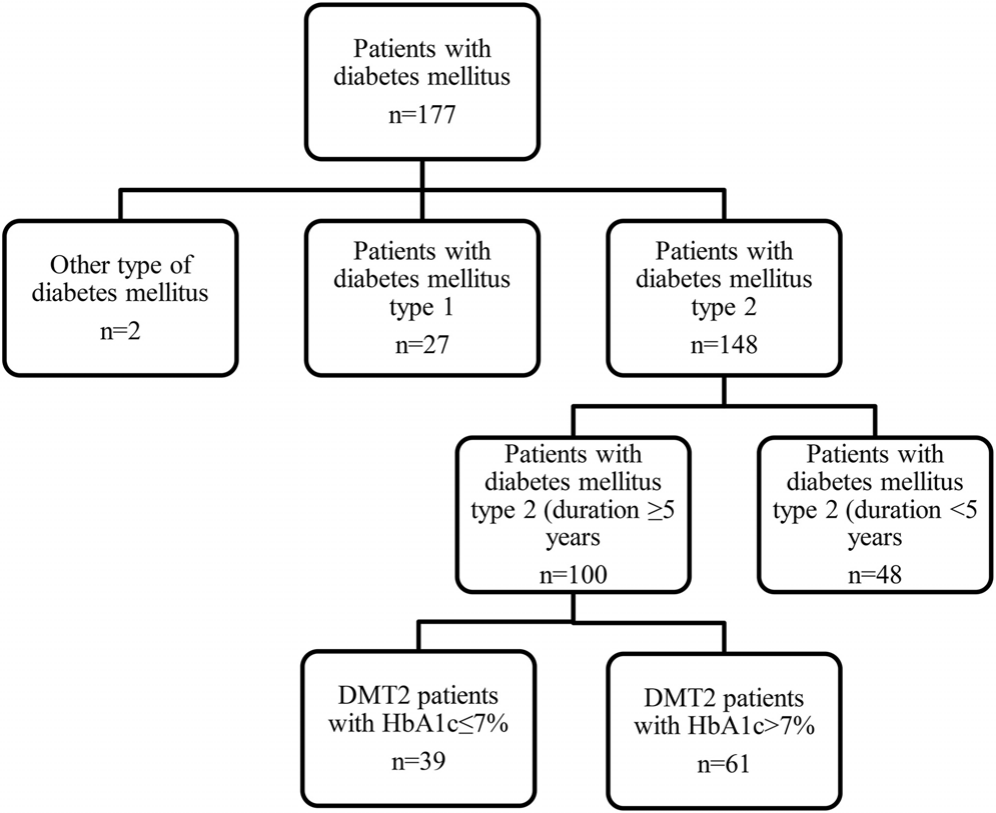
**A flowchart of patient selection.** DMT2: Diabetes mellitus type 2.

The ethics committee of the Health Center of Sarajevo Canton, Bosnia and Herzegovina, approved this study (Decision No: 01-06-33-2-4596-2/23). All patients voluntarily participated in the study. Based on plasma HbA1c levels, patients were divided into two groups: those with HbA1c ≤ 7% (*n* ═ 39) and those with HbA1c > 7% (*n* ═ 61) (Figure 1).

For this study, the following clinical characteristics were collected from patients’ medical records during office visits: age, sex, presence of hypertension, duration of DMT2, smoking status, body mass index (BMI), and complications, such as nephropathy, neuropathy, retinopathy, angina pectoris, myocardial infarction, cardiomyopathy, transient ischemic attack, and stroke.

#### Methods

Venous blood samples were collected during office visits from all patients at the time of their inclusion in the study. The samples were immediately placed into tubes containing EDTA, then centrifuged at 3000 × *g* for 10 min. The plasma was subsequently stored at −80 ^∘^C until further testing. Plasma Endocan concentrations were measured using a commercial ELISA assay (ab278119 Human Endocan SimpleStep ELISA^®^ Kit (ESM-1), Abcam, Cambridge, UK) and analyzed with the ELISA Analyzer Elisys Quattro (Human Diagnostics Worldwide, Wiesbaden, Germany). Hematological parameters (RBC, WBC, PLT, hematocrit, and hemoglobin), plasma concentrations of urea, creatinine, AST, ALT, GGT, ALP, albumin, globulin, and CRP, as well as metabolic parameters (LDL, HDL, triglycerides, cholesterol, fasting glucose level, HbA1c [%], and BMI), were measured using routine standard methods. All patients were clinically followed for one year after their inclusion in the study. During this period, all microvascular and macrovascular complications were recorded. Clinical follow-ups were conducted through office visits.

### Statistical analysis

Depending on the type of variable, continuous data are presented as means ± SD and were compared using an unpaired Student’s *t*-test. Categorical variables are reported as frequencies (%) and were compared using Fisher’s exact test. Relative risks (RRs) were calculated with 95% confidence intervals (CIs). Cox proportional hazards regression (both univariate and multivariate analyses) was employed to evaluate the predictive value of plasma endocan levels. Correlations among variables were assessed using Pearson’s correlation test. Differences were considered statistically significant if *P* < 0.05. All patient data were processed and analyzed using SPSS version 27.0 (Chicago, IL, USA).

## Results

### Clinical characteristics of the patients

This prospective observational study included 100 patients with DMT2 (mean age: 66.99 ± 8.77 years; 43 males and 57 females). Based on their HbA1c levels, the patients were divided into two groups: HbA1c ≤ 7% and HbA1c > 7%. The first step involved evaluating their hematological, metabolic parameters, and clinical characteristics. Patients with HbA1c > 7% exhibited significantly higher body BMI (31.72 ± 6.58 vs 27.85 ± 3.12, *P* ═ 0.0009), systolic blood pressure (143.51 ± 9.23 vs 133.73 ± 8.06 mmHg, *P* ═ 0.0001), diastolic blood pressure (91.74 ± 7.38 vs 79.15 ± 4.93 mmHg, *P* ═ 0.0001), fasting glucose levels (11.88 ± 3.15 vs 7.13 ± 2.07 mmol/L, *P* ═ 0.0001), triglycerides (3.17 ± 0.70 vs 2.06 ± 0.65 mmol/L, *P* ═ 0.0001), cholesterol (6.82 ± 0.36 vs 2.93 ± 0.23 mmol/L, *P* ═ 0.0001), and LDL cholesterol levels (4.73 ± 0.53 vs 1.97 ± 0.41 mmol/L, *P* ═ 0.0001). They also had significantly lower HDL cholesterol levels (0.96 ± 0.20 vs 1.52 ± 0.12 mmol/L, *P* ═ 0.0001) (Table 1). Plasma endocan levels were significantly elevated in DMT2 patients with HbA1c > 7% (1.38 ± 0.33 vs 0.68 ± 0.23 ng/mL, *P* < 0.0001) compared with those in the HbA1c ≤ 7% group (Table 2).

**Table 1:** Clinical characteristics, hematological and metabolic parameters of patients with DMT2 according to HbA1c (%)

**Variable**	**HbA1c ≤ 7%** **(*n* ═ 39)**	**HbA1c > 7%** **(*n* ═ 61)**	***P* value**
Age (years)	63.12 ± 7.39	69.46 ± 5.34	0.0001*
Body mass index	27.85 ± 3.12	31.72 ± 6.58	0.0009*
Duration of DMT2, (years)	9.47 ± 3.64	15.63 ± 2.19	0.0001*
Current smoker, *n* (%)	19 (48.72)	28 (45.9)	
Systolic blood pressure (mm Hg)	133.73 ± 8.06	143.51 ± 9.23	0.0001*
Diastolic blood pressure (mm Hg)	79.15 ± 4.93	91.74 ± 7.38	0.0001*
Fasting glucose level (mmol/L)	7.13 ± 2.07	11.88 ± 3.15	0.0001*
RBC (×10^12^)	4.86 ± 0.47	4.92 ± 0.51	0.5556
Hemoglobin (g/dL)	14.22 ± 3.43	14.83 ± 2.09	0.2714
Hematocrit (%)	37.31 ± 11.62	38.55 ± 10.44	0.5807
WBC (×10^9^)	8.37 ± 1.82	9.16 ± 2.61	0.1022
PLT (×10^9^)	268.10 ± 65.15	272.28 ± 79.11	0.7835
Urea (mmol/L)	6.36 ± 2.95	6.83 ± 2.02	0.3465
Creatinine (µmol/L)	93.67 ± 28.03	97.29 ± 15.68	0.4099
AST (UI/L)	18.72 ± 11.38	20.63 ± 9.22	0.3592
ALT (UI/L)	21.26 ± 9.75	23.92 ± 12.05	0.2501
GGT (UI/L)	30.32 ± 12.19	34.18 ± 21.03	0.3014
ALP (UI/L)	77.53 ± 15.35	81.23 ± 18.63	0.3031
Albumin (g/L)	44.81 ± 5.19	46.32 ± 4.71	0.1362
Globulin (g/L)	27.46 ± 6.05	29.33 ± 5.27	0.1057
Fibrinogen (g/L)	3.13 ± 0.82	3.48 ± 0.96	0.0631
Triglycerides (mmol/L)	2.06 ± 0.65	3.17 ± 0.70	0.0001*
Cholesterol (mmol/L)	2.93 ± 0.23	6.82 ± 0.36	0.0001*
LDL (mmol/L)	1.97 ± 0.41	4.73 ± 0.53	0.0001*
HDL (mmol/L)	1.52 ± 0.12	0.96 ± 0.20	0.0001*
CRP (mg/L)	3.43 ± 1.29	3.81 ± 1.77	0.2498

*Significant difference; Continuous variables are presented as the means ± SD and were compared with an unpaired Student’s test; Categorical variables were compared with the Fisher’s exact test. RBC: Red blood cells; WBC: White blood cells; PLT: Platelets; AST: Aminotransferase; ALT: Alanine aminotransferase; ALP: Alakaline phosphatase; GGT: Gamma glutamyl transferase; LDL: Low density lipoprotein; HDL: High density lipoprotein; CRP: C-reactive protein; DMT2: Diabetes mellitus type 2.

**Table 2:** Plasma endocan level in patients with DMT2

**Endocan (ng/mL)**	**DMT2 patients**		
	**HbA1c ≤ 7% (*n* ═ 39)**	**HbA1c **>** 7% (*n* **═** 61)**	***P***
	0.68 ± 0.23	1.38 ± 0.33	<0.0001*

DMT2 according to HbA1c (%). *Significant difference; Continuous variable is presented as the means ± SD and were compared with an unpaired Student’s test. DMT2: Diabetes mellitus type 2.

### Metabolic parameters of DMT2 patients according to endocan plasma levels

The median plasma endocan level was 1.10 ng/mL. DMT2 patients with plasma endocan levels >1.10 ng/mL demonstrated significantly higher values for several metabolic parameters: BMI (32.46 ± 2.14 vs 26.18 ± 3.04, *P* ═ 0.0001), HbA1c (%) (8.18 ± 1.69 vs 7.41 ± 1.82, *P* ═ 0.0306), fasting glucose levels (10.33 ± 4.12 vs 8.63 ± 2.58, *P* ═ 0.0161), LDL cholesterol (3.46 ± 0.72 vs 2.22 ± 0.49, *P* ═ 0.0001), total cholesterol (5.48 ± 1.63 vs 4.05 ± 2.87, *P* ═ 0.0026), and triglycerides (3.12 ± 2.46 vs 2.08 ± 0.73, *P* ═ 0.0059). In contrast, they exhibited significantly lower HDL cholesterol levels (1.11 ± 0.51 vs 1.37 ± 0.57; *P* ═ 0.0179) (Table 3).

**Table 3:** Metabolic parameters in patients with diabetes mellitus type 2 stratified according to the median of plasma endocan levels

**Metabolic parameter**	**Endocan ≤ 1.10 ng/mL (*n* **═** 44)**	**Endocan >1.10 ng/mL (*n* **═** 56)**	***P* value**
BMI	26.18 ± 3.04	32.46 ± 2.14	0.0001*
Triglycerides (mmol/L)	2.08 ± 0.73	3.12 ± 2.46	0.0059*
Cholesterol (mmol/L)	4.05 ± 2.87	5.48 ± 1.63	0.0026*
LDL (mmol/L)	2.22 ± 0.49	3.46 ± 0.72	0.0001*
HDL (mmol/L)	1.37 ± 0.57	1.11 ± 0.51	0.0179*
HbA1c (%)	7.41 ± 1.82	8.18 ± 1.69	0.0306*
Fasting glucose level (mmol/L)	8.63 ± 2.58	10.33 ± 4.12	0.0161*

*Significant difference; Continuous variables are presented as themeans ± SD and were compared with an unpaired Student’s test. BMI: Body mass index; LDL: Low density lipoprotein; HDL: High density lipoprotein.

### Risk stratification of DMT2 patients based on increased plasma endocan levels above the median value of 1.10 ng/mL

The next step was to evaluate the prognostic value of plasma endocan levels for predicting microvascular and macrovascular complications in patients with DMT2. Patients with plasma endocan levels > 1.10 ng/mL (*n* ═ 56) demonstrated significantly higher risks of developing the following complications compared to those with plasma endocan levels ≤1.10 ng/mL (*n* ═ 44) (Table 4): retinopathy: RR ═ 2.75 (95% CI: 1.22–6.22, *P* ═ 0.0152); nephropathy: RR ═ 2.10 (95% CI: 1.23–3.57, *P* ═ 0.0065); neuropathy: RR ═ 1.99 (95% CI: 1.20–3.31, *P* ═ 0.0075); angina pectoris: RR ═ 2.49 (95% CI: 1.09–5.70, *P* ═ 0.0311); hypertension: RR ═ 1.14 (95% CI: 1.01–1.29, *P* ═ 0.0398); cardiomyopathy: RR ═ 2.62 (95% CI: 1.15–5.96, *P* ═ 0.0218); myocardial infarction: RR ═ 9.43 (95% CI: 1.27–69.77, *P* ═ 0.0280); stroke: RR ═ 4.46 (95% CI: 1.38–14.48, *P* ═ 0.0127). These findings indicate that elevated plasma endocan levels ≤1.10 ng/mL are associated with a significantly increased risk of both microvascular and macrovascular complications in DMT2 patients.

**Table 4:** Risk stratification of patients with diabetes mellitus type 2 based on elevated plasma endocan level (greater than the median value 1.10 ng/mL)

**Plasma endocan level (ng/mL)**	**Overall (*n* ═ 100)**	**≤1.10 ng/mL (*n* ═ 44)**	**>1.10 ng/mL (*n* ═ 56)**	***RR (95% CI)***	****P* value***
Retinopathy, *n* (%)	27 (27)	6 (13.6)	21 (37.5)	2.7500 (1.2150–6.2244)	0.0152*
Nephropathy, *n* (%)	44 (44)	12 (27.3)	32 (57.1)	2.0952 (1.2294–3.5710)	0.0065*
Neuropathy, *n* (%)	46 (46)	13 (29.5)	33 (58.9)	1.9945 (1.2025–3.3081)	0.0075*
Angina pectoris, *n* (%)	25 (25)	6 (13.6)	19 (33.9)	2.4881 (1.0865–5.6979)	0.0311*
Hypertension, *n* (%)	93 (3)	38 (86.4)	55 (98.2)	1.1372 (1.0060–1.2856)	0.0398*
Cardiomyopathy, *n* (%)	26 (26)	6 (13.6)	20 (35.7)	2.6190 (1.1507–5.9612)	0.0218*
Myocardial infarction, *n* (%)	13 (13)	1 (2.3)	12 (21.4)	9.4286 (1.2742–69.7697)	0.0280*
Transient ischemic attack, *n* (%)	3 (3)	1 (2.3)	2 (3.6)	1.5714 (0.1472–16.7733)	0.7083*
Stroke, *n* (%)	17 (17)	3 (6.8)	14 (25.0)	4.4638 (1.3765–14.4758)	0.0127*

*Significant difference. RR: Relative risk; CI: Confidence interval.

### Correlation between plasma endocan levels and metabolic parameters in DMT2 patients

The plasma endocan level showed a significant positive correlation with HbA1c (%) (*r* ═ 0.856, *P* < 0.0001), fasting glucose levels (*r* ═ 0.631, *P* < 0.0001), BMI (*r* ═ 0.464, *P* < 0.0001), LDL cholesterol (*r* ═ 0.347, *P* ═ 0.0004), total cholesterol (*r* ═ 0.282, *P* ═ 0.0045), and triglycerides (*r* ═ 0.366, *P* ═ 0.0002). In contrast, it exhibited a significant negative correlation with HDL cholesterol (*r* ═ −0.429, *P* < 0.0001) (Table 5).

**Table 5:** The correlations between plasma endocan levels and metabolic parameters in patients with DMT2

**Endocan**		**HbA1c (%)**	**FGL**	**LDL**	**HDL**	**Cholesterol**	**Triglycerides**	**BMI**
	*r*	0.856	0.631	0.347	–0.429	0.282	0.366	0.464
	*P*	< 0.0001*	<0.0001*	0.0004*	<0.0001*	0.0045*	0.0002*	<0.0001*

*Significant difference. BMI: Body mass index; FGL: Fasting glucose level; LDL: Low density lipoprotein; HDL: High density lipoprotein; DMT2: Diabetes mellitus type 2.

## Discussion

In this study, we revealed an association between higher plasma endocan levels and poor glycemic control in DMT2 patients and increased plasma endocan levels exhibit good predictive value for the development of microvascular and macrovascular complications in these patients. To our knowledge, this is the first study evaluating predictive value of plasma endocan levels for complications in DMT2 patients.

Endocan is a relatively new biomarker of endothelial dysfunction. Its pathophysiological role and clinical relevance are not fully understood. In DM, microvascular and macrovascular complications occur due to endothelial damage. Hyperglycemia and hyperlipidemia in diabetic patients cause endothelial pathological changes and induce its dysfunction.

This study showed that the plasma endocan level was associated with poor glycemic control (HbA1c > 7%) in DMT2 patients. Plasma endocan levels are significantly increased in DMT2 patients with HbA1c > 7% compared with patients with HbA1c ≤ 7%. A systematic review and meta-analysis by Khalaji et al. [16] assessed all studies regarding serum or plasma endocan levels in individuals with prediabetes and diabetes. Several studies have analyzed endocan levels in patients with DMT2. Most of the analyzed studies found increased serum or plasma endocan levels in DMT2 patients. Arman et al. [9] reported significantly increased serum endocan concentrations in DMT2 patients compared with healthy individuals. After three months of treatment even though HbA1c decreased from 10.7% to 7.57%, the endocan level remains still higher than in healthy controls. Research done by Klisic et al. [10] showed that DMT2 patients had significantly increased serum endocan levels than healthy controls and if the endocan level increases one-fold the probability of higher HbA1c increases three-fold. However, Moin et al. [15] have reported that plasma endocan concentrations were decreased in DMT2 patients than in healthy subjects.

Among the clinical characteristics of the patients included in this study only systolic and diastolic blood pressure levels were significantly higher in DMT2 patients with higher plasma endocan levels and HbA1c > 7%. These findings are consistent with meta-analysis included several research studies showing that hypertensive patients had increased serum or plasma endocan levels [17].

In our study, the median of plasma Endocan level was 1.10 ng/mL. DMT2 patients with plasma endocan levels > 1.10 ng/mL had significantly higher values of metabolic parameters: BMI, HbA1c (%), fasting glucose level, LDL, cholesterol, triglycerides, and significantly lower value of HDL. These results are consistent with those in the research done by Klisic et al. [18] who reported opposite association between serum endocan levels and LDL and HDL in DMT2 patients.

Several studies have determined higher serum endocan levels in DMT2 patients with complications, such as neuropathy, obstructive sleep apnea (OSA), retinopathy, nephropathy, erectile dysfunction, acute coronary syndrome, heart failure, myocardial infarction, atherosclerosis, and cirrhosis compared to DMT2 without complications [19–29]. To our knowledge, this is first study determining relative risk for complications in patients with DMT2 according to the median value of endocan. The results of our study showed that patients with plasma endocan levels **>** 1.10 ng/mL had significantly higher risks of neuropathy, retinopathy nephropathy, angina pectoris, hypertension, cardiomyopathy, myocardial infarction, and brain stroke than patients with serum Endocan levels ≤1.10 ng/mL.

Recently, Klisic et al. [30] have reported the endocan level is related to increased cardiovascular risk in DMT2 patients.

A systematic review and meta-analysis of several research studies performed by Behnoush et al. [31] showed that serum or plasma endocan levels were significantly greater in patients with OSA compared with healthy subjects. In our study none of the patients had OSA. Several studies have reported that patients with COVID-19 have significantly higher serum/plasma endocan levels compared with healthy subjects [32]. No patients with COVID-19 infection were included in this research.

We found a significant positive correlation of serum endocan levels with HbA1c (%), fasting glucose level, BMI, LDL, cholesterol and triglycerides and a strong negative correlation with HDL. Klisic et al. [10] reported positive correlations between serum endocan levels and HbA1c (%), dyslipidemia, oxidative stress and inflammation and a negative correlations with HDL [18], similar to our results.

Our research has limitations that are the relatively small number of patients and it was done in only one center.

As shown in the present study, increased plasma endocan levels are associated with poor metabolic control and might be a reliable predictor for determining the risk for complications in DMT2 patients. Thus, it could help clinicians perform better risk stratification of these patients, adjusting their treatment and preventing poor clinical outcomes.

## Conclusion

An increased plasma endocan level was associated and correlated with abnormal metabolic parameters in DMT2 patients and might be good predictor of vascular and other complications in these patients.
